# Plasma Klotho concentration is associated with the presence, burden and progression of cerebral small vessel disease in patients with acute ischaemic stroke

**DOI:** 10.1371/journal.pone.0220796

**Published:** 2019-08-09

**Authors:** Ho Geol Woo, Yoonkyung Chang, Dong-Ryeol Ryu, Tae-Jin Song

**Affiliations:** 1 Department of Neurology, Ewha Womans University Seoul Hospital, Ewha Womans University College of Medicine, Seoul, Korea; 2 Department of Neurology, Ewha Womans University Mokdong Hospital, Ewha Womans University College of Medicine, Seoul, Korea; 3 Department of Nephrology, Ewha Womans University College of Medicine, Seoul, Korea; Tufts University, UNITED STATES

## Abstract

Klotho is a soluble or membrane-bound anti-aging protein, whose protective actions are important for a prudential function of many organs. Because Klotho and cerebral small vessel disease (SVD) are associated with ageing process and endothelial dysfunction, it is possible that Klotho has an association with cerebral SVD. We aimed to investigate the association of plasma Klotho concentration with the presence, burden and progression of cerebral SVD. We prospectively enrolled 262 patients with first-ever acute cerebral infarction, performed brain MRI and collected their blood samples within 24 hours of admission. An enzyme-linked immunosorbent assay was used for evaluating plasma Klotho concentration. We estimated the total SVD score of each patient after determining the presence and burden of high-grade white matter hyperintensities (HWMHs), cerebral microbleeds (CMBs), high-grade perivascular spaces (HPVSs) and asymptomatic lacunar infarctions (ALIs). Univariate and multivariate analyses were conducted to investigate association of Klotho with cerebral SVD and the total SVD score. Of the 262 patients, 152 (58.0%) were men. The mean age of these patients was 64.7 years. The mean ± standard deviation of plasma Klotho concentration was 329.8 ± 194.1 pg/mL. In multivariate analysis, plasma Klotho concentration was negatively associated with the presence of HWMHs [Odds ratio (OR): 0.13, p = 0.047], HPVSs (OR: 0.22, p = 0.024) and ALIs (OR: 0.53, p = 0.021) but not associated with the presence of CMBs (OR: 0.39, p = 0.404). Plasma Klotho concentration was also negatively related to the total SVD score (unstandardized coefficients beta: −0.895, standard error = 0.317, p = 0.005, R^2^ = 0.239). Furthermore, plasma Klotho concentration was negatively related to the presence (OR: 0.75, 95% CI: 0.59–0.96, p = 0.025) and severity of cerebral SVD progression (OR: 0.72, 95% CI: 0.56–0.92, p = 0.009). In conclusion, plasma Klotho concentration was negatively associated with the presence, burden and progression of cerebral SVD.

## Introduction

Klotho was a single-pass transmembrane protein encoded by Klotho gene that is predominantly present in the distal tubular cells of the kidney, choroid plexus of the brain and parathyroid glands [1]. The importance of this Klotho has been highlighted in the study involving the Klotho knock-out mouse model, which revealed that Klotho deficiency was related to reduced life expectancy [2] and that Klotho overexpression was associated with life span prolongation [3]. Human Klotho exists in the form of membrane Klotho and circulatory Klotho. In the kidney, membrane Klotho acts as a co-receptor for fibroblast growth factor and is involved in phosphate secretion [4], whereas circulatory Klotho plays a role in the regulation of nitric oxide in vascular endothelial cells and in calcium homeostasis [5]. Circulatory Klotho is recognized as having the enzyme activity of autocrine, endocrine and paracrine hormone. Previous clinical study demonstrated that potential link between klotho deficiency and enhanced oxidative stress in patients with kidney disease [6].

Cerebral small vessel disease (SVD) is characterized by pathologic changes in a small artery or arteriole and manifests as white matter hyperintensities (WMHs), perivascular spaces (PVSs), cerebral microbleeds (CMBs) and asymptomatic lacunar infarctions (ALIs) on specific brain MR image sequences [7]. These cerebral SVD forms share common risk factors or mechanism of development despite each forms demonstrating a different clinical impact or association; for example, i.e. WMHs, PVSs and ALIs are mainly related to cerebral ischaemia, whereas CMBs are closely associated with future cerebral haemorrhagic events [8, 9]. Because these cerebral SVD forms are closely linked with future stroke or cognitive decline events [7, 10, 11], studies to determine the associated risk factors for various cerebral SVD forms are warranted.

Previous studies using animal model were demonstrated that klotho protein protects the endothelial dysfunction through nitric oxide production by humoral pathways [12, 13]. In addition, high Klotho concentration was associated with a reduced risk of developing stroke and macroangiopathies in clinical study [14]. Furthermore, cerebral SVD is also associated with ageing process and endothelial dysfunction in human studies [15, 16]. Therefore, Klotho may have a possibility of association with cerebral SVD. In our study, we hypothesized that plasma Klotho concentration would be negatively associated with the presence, burden and progression of cerebral SVD.

## Materials and methods

### Subjects

Between June 2014 and May 2016, we prospectively enrolled 262 patients with first cerebral infarction who were admitted to our hospital within 7 days of symptom onset and whose stroke subtypes were classified as large artery atherosclerosis, cardioembolism or lacunar infarction. All patients were evaluated according to the standard protocol of our hospital, which includes 12-lead electrocardiography, chest X-ray, routine blood tests [white blood cell count and creatinine levels at admission and levels of vitamin D (25(OH)D), fasting glucose, HbA1c, triglyceride, total cholesterol, low-density lipoprotein, haemoglobin, total calcium, phosphate, albumin, alkaline phosphatase, uric acid and C-reactive protein after 12 hours fasting period] and imaging studies (CT and/or MRI, CT angiography, MR angiography or digital subtraction angiography) [17, 18]. Patients were not enrolled in the study if they did not provide consent for participation as well as when they did not provide consent for blood collection to determine plasma Klotho concentration. We excluded patients had a previous history of cancer or autoimmune disease, because impaired immune system caused by predisposing to autoimmune disease or cancer impedes an adequate control of systemic inflammation and systemic inflammation is the main downregulator of levels of Klotho [19, 20]. Furthermore, because Klotho is involved in calcium homeostasis in the kidney, we excluded patients with bone fractures in the last 2 months [21].

The definition of risk factors was established in the Supplementary methods section in a previous study (S1 Appendix) [22]. Cerebral atherosclerosis was defined as the presence of one or more vessels with over 50% stenosis or occlusion in extracranial or intracranial cerebral arteries [23]. The stroke subtype was classified according to the Trial of Org 10172 in Acute Stroke Treatment (TOAST) classification system [24]. Briefly, the diagnosis was large artery atherosclerosis if the patient had significant stenosis (≥50%) or occlusion of the relevant large artery of the lesion. Cardioembolism was diagnosed when the patient had a possible cardioembolic source. Lacunar infarction was diagnosed when the patient with acute lacunar syndrome had small (<15 mm) and deep lesion but no significant stenosis (<50%) of the relevant artery of the lesion and no possible cardioembolic source [25]. This study was approved by our Institutional Review Board (Ewha Clinical Trial Center 2014-04-023), and we received informed consents from all participants and their closest relatives.

### Brain MR protocol and definition of cerebral small vessel diseases

The detailed brain MR image sequence and definition of cerebral SVD were established in previous studies [26–28]. All subjects were examined using a 3.0 T MRI scanner (Achieva 3.0 T, Philips Medical Systems, Best, Netherlands; or MAGNETOM Trio 3.0 T, Siemens, Germany). MRI protocol, acquired along the axis of the orbitomeatal line, included T2-weighted images [time repetition (TR), 9000 ms; time echo (TE), 100 ms; field of view (FOV), 230×230 mm; slice thickness, 5 mm; pixel spacing 0.240×0.240 mm], fluid attenuated inversion recovery (FLAIR) (TR, 9000 ms; TE, 120 ms; FOV, 230×230 mm; slice thickness, 5 mm; pixel spacing, 0.449×0.449 mm), gradient echo (GRE) (TR, 600 ms; TE, 16 ms; FOV, 250×250 mm; slice thickness, 5 mm; pixel spacing, 0.449×0.449 mm) and diffusion weighted image (DWI) sequence along six different directions (x, y, z, xy, yz, zx) (TR, 2600–6500 ms; TE, 42–70 ms; FOV, 230×230 mm; slice thickness, 5 mm; intersection gap, 2 mm; b-values, 0 and 600 mm^2^/s) [28, 29].

According to Fazekas’ scoring system, the extent of WMHs was referred to as a periventricular white matter or deep white matter on FLAIR images [30]. High-grade white matter hyperintensities (HWMHs) were regarded as a Fazekas score of ≥2 in periventricular white matter and/or ≥2 in deep white matter. The PVSs were observed as punctate and/or linear hyperintense lesions located in the centrum semi-ovale or basal ganglia with a diameter of <3 mm on T2-weighted images [31]. High-grade perivascular spaces (HPVSs) were regarded as grade 2–4 PVSs based on a previous report [9]. The CMBs were regarded to be round hypointense lesions with a diameter of <10 mm on GRE images [29]. The ALIs were referred to as round or ovoid, subcortical, fluid-filled cavities (signal similar to cerebrospinal fluid) measuring 3–15 mm in diameter that manifested as hyperintense lesions on T2-weighted images and as hypointense lesions on T1-weighted images, with no history of relevant symptoms or signs. The presence of HWMHs, HPVSs, CMBs and ALIs was defined outside the acute infarcted area (based on DWI), and these lesions were independently evaluated by two neurologists (T.J.S. and Y.C.) who were blinded to the clinical information.

The total SVD score was obtained on an ordinal scale ranging from 0 to 4 by assigning a rating (0 or 1) to the presence of each of the four SVD features, namely HWMHs, HPVSs, CMBs and ALIs [32]. Inter-observer agreements for the presence of HWMHs, HPVSs, CMBs and ALIs were 0.956, 0.938, 0.912 and 0.888, respectively (all *p*<0.05) [26]. Any disagreement between the two raters regarding the presence of SVD was solved by consensus.

For evaluating the association of Klotho with cerebral SVD progression, we retrospectively included the 223 patients (85.1%: 223/262) who performed brain MRI at 2 years (± 6 months) after index stroke. Follow up brain MRI (FLAIR images, T2-weighted images and GRE images at 2 years after index stroke) is routinely recommended in our clinic for evaluating progression of cerebral SVD. The cerebral SVD progression is defined as increased total SVD score more than 1 score compared to the baseline total SVD score.

### Measurement of plasma Klotho concentration

For estimating plasma Klotho concentration, venous samples in EDTA tubes were immediately collected at admission. Plasma was separated by centrifugation (1900 g, 15 min) at 4°C [25]. The acquired plasma was stored at −80°C until analysis. An enzyme-linked immunosorbent assay (Immuno-Biological Laboratories, Gunma, Japan) was used for the evaluation of plasma Klotho concentration [33]. The detection range of this assay was 31.25–4000 pg/mL. Plasma Klotho concentration was measured in duplicate and averaged by researchers who were blinded to the clinical information (Y.C. and D.Y.R). Intra-assay and inter-assay coefficients of variability were 3.1% and 6.9%, respectively.

### Statistical analysis

Statistical analysis was performed using SPSS software package (version 21.0, Chicago, IL, USA). Continuous variables were analysed using independent t-test or Mann–Whitney test, and categorical variables were analysed using Chi-square or Fisher’s exact test. Univariate and multivariate analyses were conducted to investigate the association of Klotho with cerebral SVD. To compare Klotho concentration among stroke subtypes, Kruskal–Wallis test was performed. The variables with a p value of < 0.1 in univariate analysis, sex and age were entered into the multivariate analyses.

To investigate the relationship of Klotho with the total SVD score, univariate and multivariate linear regression analyses were performed. In model 1, sex, age, body mass index, risk factors and clinical variables (hypertension, diabetes mellitus, hypercholesterolemia, coronary artery disease, smoking, alcohol intake, pre-stroke anti-thrombotics, pre-stroke statins, cerebral atherosclerosis and stroke subtype) were adjusted. In model 2, sex, age, body mass index and blood laboratory findings (levels of fibroblast growth factor-23, vitamin D 25(OH)D, fasting glucose, HbA1c, triglyceride, total cholesterol, low-density lipoprotein, haemoglobin, creatinine, total calcium, phosphate, albumin, alkaline phosphatase, uric acid and C-reactive protein as well as white blood cell count) were adjusted. Moreover, in model 3, sex, age, body mass index and variables with a p value of < 0.1 in univariate analysis (hypertension, coronary artery disease, smoking, stroke subtype and levels of fibroblast growth factor-23, vitamin D 25(OH)D, low-density lipoprotein, creatinine and albumin) were adjusted. For convenience, intracranial and extracranial cerebral atherosclerosis were dichotomized as cerebral atherosclerosis owing to over-fitting in logistic regression and linear regression. To evaluating the association of Klotho with cerebral SVD progression, binary logistic regression for presence of cerebral SVD progression and ordinal logistic regression for severity of cerebral SVD progression were performed. The sex, age, body mass index and variables with a p value of < 0.1 in univariate analysis were entered into the multivariate analyses. A p value of < 0.05 was considered to be statistically significant.

## Results

### Demographic data and comparative analysis according to the presence of cerebral SVD

Demographic data of patients are shown in Table 1. Of the 262 patients, 152 (58.0%) were men. The mean age of the study patients was 64.7 years. The mean ± standard deviation of plasma Klotho concentration was 329.8 ± 194.1 pg/mL. Regarding stroke subtype, large artery atherosclerosis (41.2%, 108/262) was most commonly noted, followed by small vessel occlusion (40.8%) and cardioembolism (17.9%). The HWMHs were prevalent in 24.4% of subjects (64/262); HPVSs, 9.2% (24/262); CMBs, 19.5% (51/262) and ALIs, 19.5% (51/262). One hundred forty-nine (56.9%) patients had a total SVD score of 0. The total SVD score of 1 was most commonly noted (25.6%, 67/262) (Table 1). Plasma Klotho concentration did not differ according to stroke subtype (p = 0.974).

**Table 1:** Clinical characteristics of enrolled patients.

Variables	Total patients (n = 262)
Demographics	
Sex, male	152 (58.0)
Age, years	64.7 ± 12.3
Body mass index, kg/m^2^	24.0 ± 3.3
Risk factors	
Hypertension	153 (58.4)
Diabetes mellitus	108 (41.2)
Hypercholesterolaemia	74 (28.2)
Coronary artery disease	47 (17.9)
Smoking	98 (37.4)
Alcohol intake	73 (27.9)
Prior medication	
Anti-thrombotics	55 (21.0)
Statins	53 (20.2)
Cerebral atherosclerosis	125 (47.7)
Stroke subtype	
Cardioembolism	47 (17.9)
Large artery atherosclerosis	108 (41.2)
Small vessel occlusion	107 (40.8)
Cerebral small vessel disease	
High-grade white matter hyperintensities	64 (24.4)
Cerebral microbleeds	51 (19.5)
High-grade perivascular spaces	24 (9.2)
Asymptomatic lacunar infarctions	51 (19.5)
Total small vessel disease score	
0	149 (56.9)
1	67 (25.6)
2	22 (8.4)
3	17 (6.5)
4	7 (2.7)
Blood laboratory findings	
Klotho, pg/mL	329.8 ± 194.1
Fibroblast growth factor-23, pg/mL	343.7 ± 546.2
Vitamin D 25(OH)D, ng/mL	20.1 ± 6.8
Fasting glucose, mg/dL	116.2 ± 42.3
HbA1c, %	6.5 ± 1.4
Triglyceride, mg/dL	129.0 ± 95.6
Total cholesterol, mg/dL	177.5 ± 38.9
Low-density lipoprotein, mg/dL	115.0 ± 36.9
White blood cell count, ×10^3^	7.3 ± 2.4
Haemoglobin, mg/dL	13.5 ± 1.6
Creatinine, mg/dL	1.0 ± 0.8
Total calcium, mg/dL	8.2 ± 0.4
Phosphate, mg/dL	3.1 ± 0.6
Albumin, mg/dL	3.7 ± 0.3
Alkaline phosphatase, IU/L	224.4 ± 72.2
Uric acid, mg/dL	4.8 ± 1.6
C-reactive protein, mg/L	0.9 ± 2.8

Data are shown as n (%) or mean ± standard deviation

As opposed to patients without HWMHs, patients with HWMHs had significantly lower concentration of Klotho (270.5 ± 164.5 pg/mL vs. 348.9 ± 199.3 pg/mL), levels of vitamin D 25(OH)D (18.5 ± 6.4 ng/mL vs. 20.7 ± 6.9 ng/mL) and albumin (3.6 ± 2.9 mg/dL vs. 3.7 ± 3.2 mg/dL) and a higher levels of fibroblast growth factor-23 (518.6 ± 762.7 pg/mL vs. 287.2 ± 442.8 pg/mL) and haemoglobin (13.6 ± 1.5 mg/dL vs. 13.0 ± 1.7 mg/dL). The patients with HPVSs had a lower plasma Klotho concentration than those without HPVSs (241.4 ± 112.5 pg/mL vs. 338.7 ± 198.5 pg/mL). As opposed to patients without CMBs, those with CMBs had a higher prevalence of hypertension (74.5% vs. 54.5%), coronary artery disease (29.4% vs. 15.2%) and small vessel occlusion stroke subtype (56.9% vs. 37.0%). The plasma Klotho (277.2 ± 156.9 pg/mL vs. 342.5 ± 200.3 pg/mL), levels of vitamin D 25(OH)D (18.5 ± 5.5 ng/mL vs. 20.5 ± 7.0 ng/mL) and low-density lipoprotein (105.2 ± 34.9 mg/dL vs. 117.3 ± 37.0 mg/dL) were lower in patients with CMBs than in those without CMBs. The patients with ALIs had a lower plasma Klotho concentration than those without ALIs (249.9 ± 161.1 pg/mL vs. 349.1 ± 196.8 pg/mL). In contrast, patients with ALIs had higher levels of fibroblast growth factor-23 (627.0 ± 925.8 pg/mL vs. 275.2 ± 377.5 pg/mL), creatinine (1.3 ± 1.5 mg/dL vs. 0.9 ± 0.5 mg/dL) and alkaline phosphatase (249.6 ± 88.3 IU/L vs. 218.3 ± 66.5 IU/L) than those without ALIs (Table 2).

**Table 2:** Clinical characteristics and comparisons among study patients according to the presence of cerebral small vessel disease.

	HWMHs		HPVSs		CMBs		ALIs	
	(−) n = 198	(+) n = 64	(−) n = 238	(+) n = 24	(−) n = 211	(+) n = 51	(−) n = 211	(+) n = 51
Demographics								
Sex, male	123 (62.1)	29 (45.3)*	136 (57.1)	16 (66.7)	122 (57.8)	233 (59.0)	120 (56.9)	32 (62.7)
Age, years	62.5 ± 11.9	71.7 ± 11.0*	64.3 ± 12.4	68.9 ± 10.1^†^	64.3 ± 12.5	66.4 ± 11.3*	64.0 ± 12.7	67.8 ± 9.8*
BMI, kg/m^2^	24.2 ± 3.5	23.5 ± 2.8	24.0 ± 3.4	23.8 ± 2.8	24.3 ± 3.4	22.8 ± 2.6*	24.2 ± 3.5	23.1 ± 2.4*
Risk factors								
Hypertension	109 (55.1)	44 (68.8)^†^	136 (57.1)	17 (70.8)	115 (54.5)	38 (74.5)*	123 (58.3)	30 (58.8)
Diabetes mellitus	79 (39.9)	29 (45.3)	98 (41.2)	10 (41.7)	86 (40.8)	22 (43.1)	85 (40.3)	23 (45.1)
Hypercholesterolaemia	55 (27.8)	19 (29.7)	67 (28.2)	7 (29.2)	55 (26.1)	19 (37.3)	61 (28.9)	13 (25.5)
Coronary artery disease	34 (17.2)	13 (20.3)	43 (18.1)	4 (16.7)	32 (15.2)	15 (29.4)*	35 (16.6)	12 (23.5)
Smoking	82 (41.4)	16 (25.0)*	92 (38.7)	6 (25.0)	83 (39.3)	15 (29.4)	84 (39.8)	14 (27.5)
Alcohol intake	55 (27.8)	18 (28.1)	63 (26.5)	10 (41.7)	61 (28.9)	12 (23.5)	55 (26.1)	18 (35.3)
Prior medication								
Anti-thrombotics	41 (20.7)	14 (21.9)	51 (21.4)	4 (16.7)	40 (19.0)	15 (29.4)	43 (20.4)	12 (23.5)
Statins	40 (20.2)	13 (20.3)	49 (20.6)	4 (16.7)	39 (18.5)	14 (27.5)	42 (19.9)	11 (21.6)
Cerebral atherosclerosis	89 (44.9)	36 (56.3)	115 (48.3)	10 (41.7)	105 (49.8)	20 (39.2)	101 (47.9)	24 (47.1)
Stroke subtype								
Cardioembolism	30 (15.2)	17 (26.6)*	43 (18.1)	4 (16.7)	39 (18.5)	8 (15.7)*	38 (18.0)	9 (17.6)
Large artery atherosclerosis	80 (40.4)	28 (43.8)*	102 (42.9)	6 (25.0)	94 (44.5)	14 (27.5)*	88 (41.7)	20 (39.2)
Small vessel occlusion	88 (44.4)	19 (29.7)*	93 (39.1)	14 (58.3)	78 (37.0)	29 (56.9)*	85 (40.3)	22 (43.1)
Blood laboratory findings								
Klotho, pg/mL	348.9 ± 199.3	270.5 ± 164.5*	338.7 ± 198.5	241.4 ± 112.5*	342.5 ± 200.3	277.2 ± 156.9*	349.1 ± 196.8	249.9 ± 161.1*
Fibroblast growth factor-23, pg/mL	287.2 ± 442.8	518.6 ± 762.7*	318.9 ± 490.2	589.7 ± 916.2	271.6 ± 368.2	641.8 ± 935.5*	275.2 ± 377.5	627.0 ± 925.8*
Vitamin D 25(OH)D, ng/mL	20.7 ± 6.9	18.5 ± 6.4*	20.3 ± 6.9	18.1 ± 6.1	20.5 ± 7.0	18.5 ± 5.5*	20.2 ± 6.9	20.0 ± 6.5
Fasting glucose, mg/dL	113.1 ± 37.5	125.6 ± 53.8^†^	116.7 ± 42.2	110.9 ± 44.3	115.3 ± 42.0	119.8 ± 43.8	114.5 ± 40.3	123.0 ± 49.8
HbA1c, %	6.5 ± 1.4	6.6 ± 1.3	6.5 ± 1.4	6.2 ± 1.1	6.5 ± 1.4	6.5 ± 1.2	6.5 ± 1.4	6.6 ± 1.3
Triglyceride, mg/dL	129.3 ± 87.2	127.8 ± 118.6	130.0 ± 99.1	118.6 ± 48.3	124.4 ± 79.4	147.9 ± 144.1	129.5 ± 89.8	127.0 ± 117.6
Total cholesterol, mg/dL	178.0 ± 38.9	175.9 ± 39.2	178.3 ± 39.1	170.1 ± 37.1	179.2 ± 38.7	170.7 ± 39.2	178.2 ± 37.6	174.7 ± 44.1
Low-density lipoprotein, mg/dL	116.4 ± 36.6	110.6 ± 37.5	115.8 ± 36.7	106.4 ± 38.4	117.3 ± 37.0	105.2 ± 34.9*	116.7 ± 35.4	107.6 ± 42.0
White blood cell count, ×10^3^	7.3 ± 2.4	7.5 ± 2.5	7.3 ± 2.3	7.7 ± 3.0	7.4 ± 2.4	7.3 ± 2.5	7.3 ± 2.3	7.5 ± 2.7
Haemoglobin, mg/dL	13.6 ± 1.5	13.0 ± 1.7*	13.5 ± 1.6	13.3 ± 1.9	13.4 ± 1.5	13.5 ± 1.7	13.5 ± 1.6	13.3 ± 1.5
Creatinine, mg/dL	0.9 ± 0.4	1.2 ± 1.3	1.0 ± 0.8	1.0 ± 0.2	0.9 ± 0.6	1.2 ± 1.2	0.9 ± 0.5	1.3 ± 1.5*
Total calcium, mg/dL	8.2 ± 0.4	8.2 ± 0.4	8.2 ± 0.4	8.3 ± 0.4	8.2 ± 0.4	8.3 ± 0.4	8.2 ± 0.4	8.2 ± 0.4
Phosphate, mg/dL	3.1 ± 0.5	3.2 ± 0.6	3.1 ± 0.6	3.1 ± 0.4	3.1 ± 0.5	3.1 ± 0.8	3.1 ± 0.3	3.1 ± 0.7
Albumin, mg/dL	3.7 ± 3.2	3.6 ± 2.9*	3.7 ± 0.3	3.6 ± 0.2	3.7 ± 0.3	3.6 ± 0.2	3.7 ± 0.3	3.6 ± 0.3
Alkaline phosphatase, IU/L	220.2 ± 67.1	237.2 ± 85.3	223.3 ± 71.3	234.9 ± 81.1	219.6 ± 67.0	244.1 ± 88.6	218.3 ± 66.5	249.6 ± 88.3*
Uric acid, mg/dL	4.8 ± 1.5	4.9 ± 1.8	4.8 ± 1.6	4.8 ± 1.7	4.8 ± 1.5	5.1 ± 1.9	4.8 ± 1.6	4.8 ± 1.8
C-reactive protein, mg/L	0.9 ± 2.9	1.1 ± 2.1	1.0 ± 2.9	0.5 ± 0.7	1.0 ± 3.0	0.7 ± 1.7	0.9 ± 2.9	0.9 ± 2.1

Data are shown as n (%) or mean ± standard deviation.

*: *p*<0.05,

^†^: *p*<0.1.

HWMHs: high-grade white matter hyperintensities, HPVSs: high-grade perivascular spaces, CMBs: cerebral microbleeds, ALIs: asymptomatic lacunar infarctions, BMI: body mass index.

### Association between plasma Klotho concentration and cerebral small vessel disease

**Fig 1:**
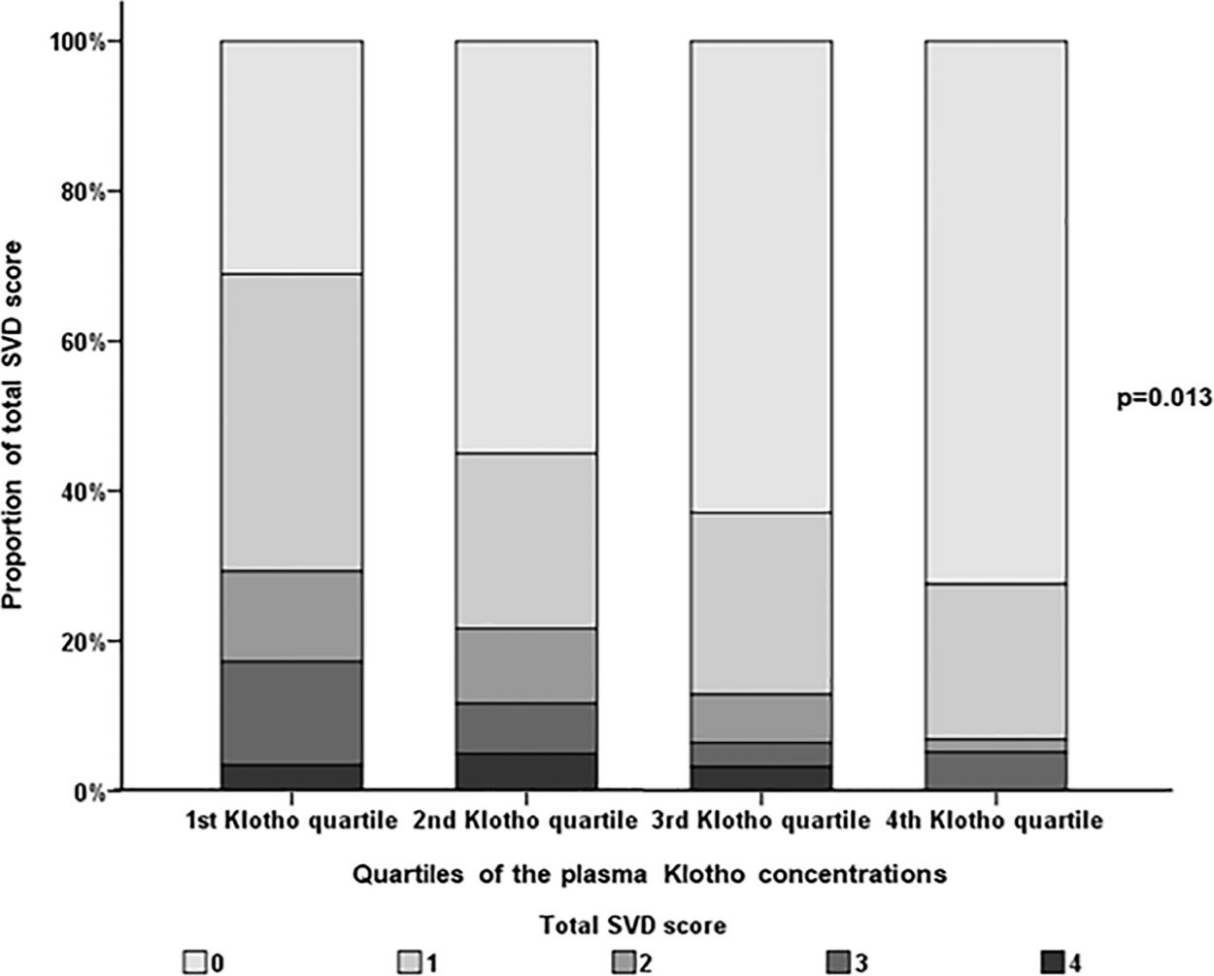
Number of total SVD score stratified by quartiles of the plasma Klotho concentration.

Considering the plasma Klotho concentration, the study population was categorized into quartiles according to the plasma Klotho concentration (136.2 ± 49.7 pg/mL, 248.1 ± 25.1 pg/mL, 347.4 ± 28.8 pg/mL and 588.7 ± 190.8 pg/mL). Cerebral atherosclerosis, presence of HWMHs and ALIs, total SVD score and fibroblast growth factor-23 were significantly different according to quartiles of the plasma Klotho concentration (S1 Table and Fig 1). In multivariate analysis, plasma Klotho concentration was negatively associated with the presence of HWMHs [Odds ratio (OR): 0.13, 95% confidence interval (CI): 0.01–0.97, p = 0.047)], HPVSs (OR: 0.22, 95% CI: 0.10–0.60, p = 0.024), and ALIs (OR: 0.53, 95% CI: 0.40–0.64, p = 0.021) but not with the presence of CMBs (OR: 0.39, 95% CI: 0.04–3.46, p = 0.404) (Table 3). No statistically significant interaction of Klotho with demographics, risk factors and blood laboratory findings were noted with regard to the presence of HWMHs, HPVSs, CMBs and ALIs.

**Table 3:** Multivariate analysis for the presence of cerebral small vessel disease.

	Presence of cerebral small vessel disease			
	HWMHs^a^	HPVSs^b^	CMBs^c^	ALIs^d^
Sex	0.98 (0.44–2.17)	2.37 (0.88–6.34)^†^	1.21 (0.57–2.56)	2.13 (1.00–4.57)^†^
Age	1.07 (1.03–1.11)*	1.04 (1.00–1.08)*	1.00 (0.96–1.04)	1.02 (0.99–1.05)
Body mass index			0.83 (0.73–0.94)*	0.89 (0.80–1.00)^†^
Hypertension	1.27 (0.63–2.54)		2.45 (1.11–5.44)*	
Coronary artery disease			2.01 (0.87–4.66)	
Smoking	0.78 (0.34–1.76)			
Stroke subtype				
SVO	1.01 (0.47–2.18)		3.79 (1.62–8.86)*	
LAA	1.18 (0.49–2.80)		1.34 (0.44–4.04)	
CE	Reference	Reference	Reference	Reference
Klotho	0.13 (0.01–0.97)*	0.22 (0.10–0.60)*	0.39 (0.04–3.46)	0.53 (0.40–0.64)*
Fibroblast growth factor-23	1.22 (0.70–2.11)		2.85 (1.35–5.99)*	1.86 (0.99–3.52)^†^
Vitamin D 25(OH)D	0.96 (0.91–1.01)		0.97 (0.92–1.03)	
Fasting glucose	1.01 (1.00–1.02)*			
Low-density lipoprotein			0.98 (0.97–1.00)^†^	
Haemoglobin	1.03 (0.80–1.32)			
Creatinine				1.22 (0.84–1.79)
Albumin	0.50 (0.16–1.57)			
Alkaline phosphatase				1.00 (1.00–1.01)*

Data are shown as odds ratio (95% confidence interval)

*: *p*<0.05,

^†^: *p*<0.1.

HWMHs: high-grade white matter hyperintensities, HPVSs: high-grade perivascular spaces, CMBs: cerebral microbleeds, ALIs: asymptomatic lacunar infarctions, SVO: Small vessel occlusion, LAA: Large artery atherosclerosis, CE: Cardioembolism

^a^ adjusted for sex, age, hypertension, smoking, stroke subtype and levels of fibroblast growth factor-23, vitamin D 25(OH)D, fasting glucose, haemoglobin, and albumin.

^b^ adjusted for sex and age.

^c^ adjusted for sex, age, BMI, hypertension, coronary artery disease, stroke subtype and levels of fibroblast growth factor-23, vitamin D 25(OH)D, and low-density lipoprotein.

^d^ adjusted for sex, age, BMI and levels of fibroblast growth factor-23, creatinine, and alkaline phosphatase.

Considering the total SVD score, the study population was categorized into total SVD score. Age, plasma Klotho concentration, fibroblast growth factor-23, fasting glucose and creatinine were significantly different according to total SVD score (S2 Table). In multivariate analysis, plasma Klotho concentration was negatively related to the total SVD score in model 1 (unstandardized coefficients beta: −1.314, standard error = 0.321, p = 0.001, R^2^ = 0.199), model 2 (unstandardized coefficients beta: −0.888, standard error = 0.329, p = 0.007, R^2^ = 0.260) and model 3 (unstandardized coefficients beta: −0.895, standard error = 0.317, p = 0.005, R^2^ = 0.239) (Table 4).

**Table 4:** Association of plasma Klotho with total SVD score.

	Unstandardized coefficients		Standardized coefficients beta	T	*p* value	R^2^
	B	SE				
Univariate	−1.288	0.322	−0.240	−3.994	0.001	0.058
Multivariate						
Model 1	−1.314	0.321	−0.245	−4.094	0.001	0.199
Model 2	−0.888	0.329	−0.166	−2.703	0.007	0.260
Model 3	−0.895	0.317	−0.167	−2.828	0.005	0.239

SVD: small vessel disease, SE: Standard error.

Model 1: adjusted for sex, age, body mass index, risk factors and clinical variables (hypertension, diabetes mellitus, hypercholesterolemia, coronary artery disease, smoking, alcohol intake, pre-stroke anti-thrombotics, pre-stroke statins, cerebral atherosclerosis, and stroke subtype)

Model 2: adjusted for sex, age, body mass index and blood laboratory findings (levels of fibroblast growth factor-23, vitamin D 25(OH)D, fasting glucose, HbA1c, triglyceride, total cholesterol, low-density lipoprotein, haemoglobin, creatinine, total calcium, phosphate, albumin, alkaline phosphatase, uric acid, and C-reactive protein as well as white blood cell count)

Model 3: adjusted for sex, age, body mass index and variables with a p value of <0.1 in univariate analysis (hypertension, coronary artery disease, smoking, stroke subtype and levels of fibroblast growth factor-23, vitamin D 25(OH)D, low-density lipoprotein, creatinine, and albumin)

Number of total SVD score was lowest in patients with 4th quartile of the plasma Klotho concentration, followed by those with 3rd quartile of the plasma Klotho concentration, those with 2nd quartile of the plasma Klotho concentration, compared with those with 1st quartile of the plasma Klotho concentration (p = 0.013).

### Association between plasma Klotho concentration and cerebral small vessel disease progression

Among 223 patients who performed follow up brain MRI, 58 (26.0%) patients showed increased total SVD score [36 (16.1%) patients were increased with one point, 11 (4.9%) patients were increased with two points, 7 (3.1%) patients were increased with three points and 4 (1.8%) patients were increased with four points]. The patients with cerebral SVD progression had significantly old age (68.4 ± 8.8 years vs. 62.5 ± 12.8 years), higher prevalence of hypertension (72.4% vs. 53.3%) and lower level of plasma Klotho (251.8 ± 146.3 pg/mL vs. 354.3 ± 202.7 pg/mL) than those without cerebral SVD progression (S3 Table). Considering the relationship of Klotho with cerebral SVD progression, plasma Klotho concentration was negatively related with cerebral SVD progression (presence of progression; OR: 0.75, 95% CI: 0.59–0.96, p = 0.025, severity of progression; OR: 0.72, 95% CI: 0.56–0.92, p = 0.009) (Table 5).

**Table 5:** Multivariate analysis for the progression of cerebral small vessel disease.

	Presence of cerebral SVD progression^a^	Burden of cerebral SVD progression^b^
Sex	1.32 (0.63–2.73)	1.08 (0.54–2.15)
Age	1.03 (0.99–1.07)^†^	1.03 (0.99–1.06)^†^
Body mass index	0.94 (0.84–1.06)	0.96 (0.84–1.06)
Hypertension	1.92 (0.93–3.99)^†^	1.84 (0.91–3.78)^†^
Klotho	0.75 (0.59–0.96)*	0.72 (0.56–0.92)*
FGF-23	1.07 (0.99–1.14)^†^	1.05 (1.00–1.10)*
Total cholesterol	0.99 (0.98–1.01)	0.99 (0.98–1.01)
Low-density lipoprotein	0.99 (0.97–1.00)	0.99 (0.98–1.01)
White blood cell count	1.06 (0.93–1.21)	1.04 (0.92–1.17)
Total calcium	1.85 (0.78–4.39)	1.46 (0.66–3.22)

Data are shown as odds ratio (95% confidence interval)

*: *p*<0.05,

^†^: *p*<0.1. FGF-23: fibroblast growth factor-23.

^a^: presence of~: any increase in total SVD score.

^b^: burden of ~: increased point in total SVD score.

## Discussion

To date, the association of Klotho with cerebral SVD has been rarely reported. In previous studies involving patients with cognitive dysfunction, reduced plasma Klotho level was independently correlated with the degree of cerebral WMHs and was accompanied by cognitive decline [34], especially vascular dementia [35]. In a previous study which dealt with Klotho and vascular surrogate markers or cardiovascular outcome, low level of Klotho was found to be associated with the presence and severity of coronary artery disease [36]. Moreover, in patients with renal dysfunction, decreased level of circulating Klotho was correlated with brachial-ankle pulse wave velocity which represented arterial stiffness [37]. In patients who received dialysis therapy, decreased serum Klotho level was associated with cardiovascular morbidity and mortality [38]. In line with results of these previous studies, the key finding of our study is that 1) plasma Klotho concentration was negatively associated with the presence of cerebral SVD, such as HWMHs, HPVSs, CMBs (even though this was noted in univariate analysis only) and ALIs, 2) plasma Klotho concentration was negatively correlated an increased burden of cerebral SVD and 3) plasma Klotho concentration was also negatively related with progression of cerebral SVD in stroke patients.

Although our study did not suggest the exact mechanism of underlying the relationship between plasma Klotho and cerebral SVD, several hypotheses can explain this association. First, endothelial dysfunction may explain correlation of Klotho and cerebral SVD [15], because Klotho may have vasoprotective effects on the endothelium and involve in vasorelaxation that reduces endothelial dysfunction via nitric oxide pathway [13, 39] and endothelial dysfunction is associated with cerebral SVD [40]. Second, reactive oxygen species may explain correlation of Klotho and cerebral SVD, because Klotho is involved in vascular protection by inhibiting the production of reactive oxygen species and a previous study has suggested that reactive oxygen species including oxidative stress are closely related to cerebral SVD [41, 42]. Third, Klotho inhibited expression of adhesion molecules, pro-inflammatory, and pro-thrombotic factors which are concerned with cell apoptosis and inflammation [43]. Because those factors are associated with oxidative stress, relationship between plasma Klotho and cerebral SVD may be explained.

In our study, the presence of CMBs was not independently associated with plasma Klotho concentration despite showing an association in univariate analysis. The reason for this result may be that the sample size of our study may be small to indicate statistical significance and that other factors, such as hypertension, are more strongly associated with CMBs than Klotho. Additionally, because cerebral SVD such as HWMHs, HPVs and ALIs is more closely linked with cerebral ischaemia mechanisms than CMBs, which are more closely related to the cerebral bleeding mechanism, Klotho may be more closely related to ischaemia or infarction-related mechanisms than brain haemorrhage. Further research is warranted in this context.

This study had limitations. We did not investigate blood samples from the general population. However, the main goals of our study were to demonstrate the association of the presence, burden, and progression of cerebral SVD. Second, all of our blood samples and brain image findings were acquired from patients with acute stroke at the time of admission. Therefore, we were unable to investigate serial changes in Klotho concentration and progression in cerebral SVD findings of brain MRI. Third, we did not perform lumbar puncture for CSF study, although Klotho which is present in choroid plexus might be good to test for association Klotho with cerebral SVD. The reason was because lumbar puncture for CSF study was risky procedure in acute ischemic stroke patients because patients was taking medication including anti-thrombotic agent.

## Conclusions

Our study demonstrated that plasma Klotho concentration was negatively associated with the presence, burden and progression of cerebral SVD in patients with first-ever ischaemic stroke. We attribute these associations to the pleiotropic roles of Klotho in cerebral SVD.

## Supporting information

S1 Dataset Open data extraction. (XLS)

S1 Appendix Supplementary methods. (DOCX)

S2 Appendix STROBE statement. Checklist of items that should be included in reports of observational studies.
(DOC)

S1 Table Characteristics of the study subjects according to plasma Klotho concentration quartile. (DOCX)

S2 Table Characteristics of the study subjects according to total small vessel disease score. (DOCX)

S3 Table Characteristics of the study subjects according to progression of cerebral small vessel disease. (DOCX)
