# Supplementary material for: Plasma Klotho concentration is associated with the presence, burden and progression of cerebral small vessel disease in patients with acute ischaemic stroke
Source: PLoS One. 2019 Aug 9;14(8):e0220796. doi: 10.1371/journal.pone.0220796 (PMC6688787; doi:10.1371/journal.pone.0220796)
Supplement: S1 Appendix — (DOCX) [file pone.0220796.s002.docx]

**Supplementary Methods**

**Clinical variables**

A detailed definition of risk factors for hypertension was described in a previous study [1]. In brief, hypertension was defined as being present when a patient had been taking blood pressure-lowering agents, or had a resting systolic blood pressure ≥140 mmHg or diastolic blood pressure ≥90 mmHg on repeated measurements. Diabetes mellitus was diagnosed when the patient had a fasting blood glucose level ≥7.0 mmol/L, or was being treated with oral glucose-lowering medications or insulin. Hypercholesterolaemia was diagnosed if the patient had total cholesterol ≥6.2 mmol/L, low-density lipoprotein cholesterol ≥4.1 mmol/L, or if the patient had taken lipid-lowering medications after a diagnosis of hyperlipidemia. Coronary artery disease was defined as a history of myocardial infarction, unstable angina, or angiographically confirmed coronary artery occlusive disease. Patients were defined as smokers if they were current smokers or had stopped smoking within 1 year before the index stroke. Subjects whose recent mean weekly alcohol intake had regularly exceeded 300 g of ethanol were classified as heavy drinkers [2].

**References**

1. Song TJ, Park JH, Choi KH, Kim JH, Choi Y, Chang Y, et al. Is obstructive sleep apnea associated with the presence of intracranial cerebral atherosclerosis? Sleep & breathing = Schlaf & Atmung. 2017;21(3):639-46. doi: 10.1007/s11325-016-1450-9. PubMed PMID: 28168435.

2. Song TJ, Kim YD, Yoo J, Kim J, Chang HJ, Hong GR, et al. Association between Aortic Atheroma and Cerebral Small Vessel Disease in Patients with Ischemic Stroke. J Stroke. 2016;18(3):312-20. doi: 10.5853/jos.2016.00171. PubMed PMID: 27488980; PubMed Central PMCID: PMC5066433.
