# Supplementary material for: Plasma Klotho concentration is associated with the presence, burden and progression of cerebral small vessel disease in patients with acute ischaemic stroke
Source: PLoS One. 2019 Aug 9;14(8):e0220796. doi: 10.1371/journal.pone.0220796 (PMC6688787; doi:10.1371/journal.pone.0220796)
Supplement: S1 Table — (DOCX) [file pone.0220796.s004.docx]

**S1 Table.** Characteristics of the study subjects according to plasma Klotho concentration quartile

|  | Klotho quartile | | | | *p* value |
| --- | --- | --- | --- | --- | --- |
|  | Quartile 1 (n =65) | Quartile 2 (n =66) | Quartile 3 (n =66) | Quartile 4 (n =65) |  |
| Plasma Klotho, pg/mL | 136.2 ± 49.7 | 248.1 ± 25.1 | 347.4 ± 28.8 | 588.7 ± 190.8 |  |
| Demographics |  |  |  |  |  |
| Sex, male | 34 (52.3) | 41 (62.1) | 38 (57.6) | 39 (60.0) | 0.697 |
| Age, years | 67.4 ± 13.6 | 63.8 ± 11.5 | 63.7 ± 11.8 | 64.1 ± 12.2 | 0.251 |
| Body mass index, kg/m^2^ | 23.7 ± 3.1 | 24.5 ± 3.7 | 23.8 ± 3.3 | 24.3 ± 3.4 | 0.479 |
| Risk factors |  |  |  |  |  |
| Hypertension | 39 (60.0) | 34 (51.5) | 42 (63.6) | 38 (58.5) | 0.552 |
| Diabetes mellitus | 26 (40.0) | 30 (45.5) | 27 (40.9) | 25 (38.5) | 0.865 |
| Hypercholesterolaemia | 19 (29.2) | 15 (22.7) | 24 (36.4) | 16 (24.6) | 0.309 |
| Coronary artery disease | 15 (23.1) | 12 (18.2) | 10 (15.2) | 10 (15.4) | 0.614 |
| Smoking | 22 (33.8) | 27 (40.9) | 23 (34.8) | 26 (40.0) | 0.785 |
| Alcohol intake | 20 (30.8) | 23 (34.8) | 19 (28.8) | 11 (16.9) | 0.123 |
| Prior medication |  |  |  |  |  |
| Anti-thrombotics | 17 (26.2) | 16 (24.2) | 11 (16.7) | 11 (16.9) | 0.414 |
| Statins | 13 (20.0) | 14 (21.2) | 12 (18.2) | 14 (21.5) | 0.963 |
| Cerebral atherosclerosis | 43 (66.2) | 32 (48.5) | 27 (40.9) | 23 (35.4) | 0.003 |
| Stroke subtype |  |  |  |  | 0.381 |
| Cardioembolism | 14 (21.5) | 9 (13.6) | 10 (15.2) | 14 (21.5) |  |
| Large artery atherosclerosis | 30 (46.2) | 23 (34.8) | 30 (45.5) | 25 (38.5) |  |
| Small vessel occlusion | 21 (32.3) | 34 (51.5) | 26 (39.4) | 26 (40.0) |  |
| Cerebral small vessel disease |  |  |  |  |  |
| High-grade white matter hyperintensities | 21 (32.3) | 20 (30.3) | 15 (22.7) | 8 (12.3) | 0.034 |
| Cerebral microbleeds | 19 (29.2) | 11 (16.7) | 11 (16.7) | 10 (15.4) | 0.151 |
| High-grade perivascular spaces | 8 (12.3) | 9 (13.6) | 5 (7.6) | 2 (3.1) | 0.141 |
| Asymptomatic lacunar infarctions | 23 (35.4) | 11 (16.7) | 12 (18.2) | 5 (7.7) | 0.001 |
| Total small vessel disease score |  |  |  |  | 0.013 |
| 0 | 23 (35.4) | 38 (57.6) | 40 (60.6) | 48 (73.8) |  |
| 1 | 25 (38.5) | 15 (22.7) | 15 (22.7) | 12 (18.5) |  |
| 2 | 7 (10.8) | 6 (9.1) | 7 (10.6) | 2 (3.1) |  |
| 3 | 8 (12.3) | 4 (6.1) | 2 (3.0) | 3 (4.6) |  |
| 4 | 2 (3.1) | 3 (4.5) | 2 (3.0) | 0 (0.0) |  |
| Blood laboratory findings |  |  |  |  |  |
| Fibroblast growth factor-23, pg/ml | 781.0 ± 923.0 | 198.1 ± 264.4 | 206.5 ± 136.5 | 193.7 ± 128.5 | <0.001 |
| Vitamin D 25(OH)D, ng/mL | 19.0 ± 6.4 | 20.9 ± 7.8 | 20.3 ± 6.7 | 20.4 ± 6.4 | 0.417 |
| Fasting glucose, mg/dL | 114.0 ± 36.5 | 118.8 ±43.5 | 126.0 ± 55.0 | 105.9 ± 28.0 | 0.050 |
| HbA1c, % | 6.3 ± 1.0 | 6.5 ± 1.3 | 6.8 ±1 .9 | 6.6 ± 1.3 | 0.451 |
| Triglyceride, mg/dL | 140.5 ± 91.6 | 117.4 ± 67.0 | 132.1 ± 89.8 | 126.1 ± 126.0 | 0.568 |
| Total cholesterol, mg/dL | 180.4 ± 43.0 | 172.8 ± 35.4 | 181.5 ± 41.8 | 175.6 ± 35.1 | 0.541 |
| Low-density lipoprotein, mg/dL | 115.6 ± 42.9 | 113.0 ± 32.3 | 118.4 ± 39.5 | 113.0 ± 32.4 | 0.810 |
| White blood cell count, ×10^3^ | 7.8 ± 2.7 | 7.7 ± 2.8 | 7.0 ± 1.9 | 7.0 ± 2.2 | 0.071 |
| Haemoglobin, mg/dL | 13.2 ± 1.7 | 13.6 ± 1.5 | 13.4 ± 1.5 | 13.8 ± 1.7 | 0.206 |
| Creatinine, mg/dL | 1.2 ± 1.2 | 0.9 ± 0.2 | 1.0 ± 1.1 | 0.9 ± 0.2 | 0.078 |
| Total calcium, mg/dL | 8.3 ± 0.4 | 8.3 ± 0.4 | 8.3 ± 0.4 | 8.3 ± 0.5 | 0.915 |
| Phosphate, mg/dL | 3.2 ± 0.7 | 3.2 ± 0.6 | 3.1 ± 0.6 | 3.2 ± 0.6 | 0.919 |
| Albumin, mg/dL | 3.6 ± 0.4 | 3.7 ± 0.3 | 3.7 ± 0.3 | 3.7 ± 0.3 | 0.336 |
| Alkaline phosphatase, IU/L | 219.4 ± 73.6 | 220.6 ± 74.0 | 235.1 ± 72.8 | 222.5 ± 68.8 | 0.580 |
| Uric acid, mg/dL | 5.0 ± 2.1 | 5.2 ± 1.6 | 4.6 ± 1.6 | 4.7 ± 1.1 | 0.119 |
| C-reactive protein, mg/L | 1.2 ± 2.2 | 0.6 ± 1.3 | 0.8 ± 1.8 | 1.3 ± 4.7 | 0.504 |
