# Supplementary material for: Plasma Klotho concentration is associated with the presence, burden and progression of cerebral small vessel disease in patients with acute ischaemic stroke
Source: PLoS One. 2019 Aug 9;14(8):e0220796. doi: 10.1371/journal.pone.0220796 (PMC6688787; doi:10.1371/journal.pone.0220796)
Supplement: S2 Table — (DOCX) [file pone.0220796.s005.docx]

**S2 Table.** Characteristics of the study subjects according to total small vessel disease score

|  | Total small vessel disease score | | | | | *p* value |
| --- | --- | --- | --- | --- | --- | --- |
|  | 0 (n = 149) | 1 (n = 67) | 2 (n = 22) | 3 (n = 17) | 4 (n = 7) |  |
| Demographics |  |  |  |  |  |  |
| Sex, male | 88 (59.1) | 40 (59.7) | 10 (45.5) | 9 (52.9) | 5 (71.4) | 0.687 |
| Age, years | 62.1 ± 12.6 | 67.5 ± 11.0 | 68.4 ± 13.0 | 71.6 ± 8.4 | 68.3 ± 11.6 | 0.001 |
| Body mass index, kg/m^2^ | 24.4 ± 3.8 | 23.8 ± 2.6 | 24.0 ± 3.1 | 22.2 ± 2.5 | 22.9 ± 2.5 | 0.092 |
| Risk factors |  |  |  |  |  |  |
| Hypertension | 78 (52.3) | 44 (65.7) | 13 (59.1) | 13 (76.5) | 5 (71.4) | 0.166 |
| Diabetes mellitus | 58 (38.9) | 28 (41.8) | 12 (54.5) | 8 (47.1) | 2 (28.6) | 0.619 |
| Hypercholesterolaemia | 40 (26.8) | 19 (28.4) | 6 (27.3) | 9 (52.9) | 0 (0.0) | 0.091 |
| Coronary artery disease | 21 (14.1) | 14 (20.9) | 6 (27.3) | 6 (35.3) | 0 (0.0) | 0.084 |
| Smoking | 64 (43.0) | 24 (35.8) | 5 (22.7) | 3 (17.6) | 2 (28.6) | 0.130 |
| Alcohol intake | 39 (26.2) | 21 (31.3) | 5 (22.7) | 5 (29.4) | 3 (42.9) | 0.789 |
| Prior medication |  |  |  |  |  |  |
| Anti-thrombotics | 28 (18.8) | 15 (22.4) | 6 (27.3) | 6 (35.3) | 0 (0.0) | 0.288 |
| Statins | 28 (18.8) | 15 (22.4) | 4 (18.2) | 5 (29.4) | 1 (14.3) | 0.830 |
| Cerebral atherosclerosis | 69 (46.3) | 34 (50.7) | 12 (54.5) | 8 (47.1) | 2 (28.6) | 0.771 |
| Stroke subtype |  |  |  |  |  | 0.159 |
| Cardioembolism | 23 (15.4) | 15 (22.4) | 4 (18.2) | 5 (29.4) | 0 (0.0) |  |
| Large artery atherosclerosis | 70 (47.0) | 19 (28.4) | 10 (45.5) | 7 (41.2) | 2 (28.6) |  |
| Small vessel occlusion | 56 (37.6) | 33 (49.3) | 8 (36.4) | 5 (29.4) | 5 (71.4) |  |
| Cerebral small vessel disease |  |  |  |  |  |  |
| High-grade white matter hyperintensities | 0 (0.0) | 25 (37.3) | 16 (72.7) | 16 (94.1) | 7 (100.0) | <0.001 |
| Cerebral microbleeds | 0 (0.0) | 21 (31.3) | 8 (36.4) | 15 (88.2) | 7 (100.0) | <0.001 |
| High-grade perivascular spaces | 0 (0.0) | 5 (7.5) | 6 (27.3) | 6 (35.3) | 7 (100.0) | <0.001 |
| Asymptomatic lacunar infarctions | 0 (0.0) | 16 (23.9) | 14 (63.6) | 14 (82.4) | 7 (100.0) | <0.001 |
| Blood laboratory findings |  |  |  |  |  |  |
| Plasma Klotho, pg/mL | 376.2 ± 211.5 | 271.9 ± 131.1 | 283.9 ± 176.6 | 252.5 ± 192.3 | 229.1 ± 107.9 | <0.001 |
| Fibroblast growth factor-23, pg/ml | 250.4 ± 370.9 | 303.7 ± 305.0 | 430.3 ± 509.0 | 1116.8 ± 1371.6 | 565.1 ± 790.6 | <0.001 |
| Vitamin D 25(OH)D, ng/mL | 21.2 ± 7.0 | 18.7 ± 6.5 | 19.3 ± 7.2 | 18.2 ± 5.3 | 20.2 ± 5.9 | 0.076 |
| Fasting glucose, mg/dL | 112.6 ± 37.6 | 115.5 ± 41.9 | 140.6 ± 60.5 | 128.8 ± 51.3 | 92.9 ± 23.0 | 0.016 |
| HbA1c, % | 6.6 ± 1.6 | 6.3 ± 1.1 | 6.7 ± 1.2 | 7.0 ± 1.7 | 6.2 ± 1.3 | 0.431 |
| Triglyceride, mg/dL | 127.8 ± 82.4 | 129.2 ± 102.3 | 118.4 ± 62.0 | 165.4 ± 189.8 | 98.0 ± 23.2 | 0.482 |
| Total cholesterol, mg/dL | 181.5 ± 37.0 | 172.4 ± 41.4 | 166.2 ± 40.6 | 177.0 ± 47.3 | 181.1 ± 20.5 | 0.332 |
| Low-density lipoprotein, mg/dL | 119.7 ± 35.5 | 110.9 ± 36.9 | 106.9 ± 41.2 | 100.2 ± 42.5 | 116.0 ± 28.5 | 0.136 |
| White blood cell count, ×10^3^ | 7.5 ± 2.6 | 6.9 ± 1.8 | 7.0 ± 1.8 | 7.9 ± 2.6 | 9.2 ± 4.7 | 0.062 |
| Haemoglobin, mg/dL | 13.6 ± 1.5 | 13.5 ± 1.8 | 13.2 ± 1.8 | 13.0 ± 1.8 | 13.5 ± 1.4 | 0.589 |
| Creatinine, mg/dL | 0.9 ± 0.2 | 1.1 ± 0.9 | 1.3 ± 1.8 | 1.5 ± 1.6 | 1.2 ± 0.3 | 0.010 |
| Total calcium, mg/dL | 8.3 ± 0.4 | 8.3 ± 0.4 | 8.1 ± 0.5 | 8.4 ± 0.5 | 8.4 ± 0.3 | 0.347 |
| Phosphate, mg/dL | 3.2 ± 0.6 | 3.1 ± 0.6 | 3.1 ± 0.4 | 3.3 ± 1.1 | 3.0 ± 0.6 | 0.570 |
| Albumin, mg/dL | 3.7 ± 0.3 | 3.7 ± 0.3 | 3.6 ± 0.3 | 3.6 ± 0.3 | 3.7 ± 0.3 | 0.370 |
| Alkaline phosphatase, IU/L | 215.5 ± 61.6 | 228.1 ± 76.7 | 240.5 ± 86.3 | 253.8 ± 107.9 | 256.9 ± 68.8 | 0.100 |
| Uric acid, mg/dL | 4.9 ± 1.5 | 4.8 ± 1.8 | 4.6 ± 1.7 | 5.1 ±2 .1 | 5.5 ± 2.2 | 0.716 |
| C-reactive protein, mg/L | 1.0 ± 3.4 | 0.8 ± 1.6 | 0.9 ± 1.8 | 1.3 ± 2.7 | 0.4 ± 0.8 | 0.945 |
