# Supplementary material for: Plasma Klotho concentration is associated with the presence, burden and progression of cerebral small vessel disease in patients with acute ischaemic stroke
Source: PLoS One. 2019 Aug 9;14(8):e0220796. doi: 10.1371/journal.pone.0220796 (PMC6688787; doi:10.1371/journal.pone.0220796)
Supplement: S3 Table — (DOCX) [file pone.0220796.s006.docx]

**S3 Table.** Characteristics of the study subjects according to progression of cerebral small vessel disease

|  | Total | Progression of cerebral small vessel disease | | *p* value |
| --- | --- | --- | --- | --- |
|  | (n = 223) | (-) (n = 165) | (+) (n = 58) |  |
| Demographics |  |  |  |  |
| Sex, male | 131 (58.7) | 99 (60.0) | 32 (55.2) | 0.521 |
| Age, years | 64.7 ± 12.3 | 62.5 ± 12.8 | 68.4 ± 8.8 | <0.001 |
| Body mass index, kg/m^2^ | 24.0 ± 3.3 | 24.1 ± 3.4 | 23.7 ± 2.7 | 0.379 |
| Risk factors |  |  |  |  |
| Hypertension | 130 (58.3) | 88 (53.3) | 42 (72.4) | 0.011 |
| Diabetes mellitus | 93 (41.7) | 67 (40.6) | 26 (44.8) | 0.575 |
| Hypercholesterolaemia | 65 (29.1) | 49 (29.7) | 16 (27.6) | 0.761 |
| Coronary artery disease | 40 (17.9) | 30 (18.2) | 10 (17.2) | 0.872 |
| Smoking | 84 (37.7) | 66 (40.0) | 18 (31.0) | 0.225 |
| Alcohol intake | 63 (28.3) | 42 (25.5) | 21 (36.2) | 0.118 |
| Prior medication |  |  |  |  |
| Anti-thrombotics | 48 (21.5) | 34 (20.6) | 14 (24.1) | 0.573 |
| Statins | 46 (20.6) | 36 (21.8) | 10 (17.2) | 0.459 |
| Cerebral atherosclerosis | 106 (47.5) | 76 (46.1) | 30 (51.7) | 0.458 |
| Stroke subtype |  |  |  | 0.880 |
| Cardioembolism | 34 (15.2) | 24 (14.5) | 10 (17.2) |  |
| Large artery atherosclerosis | 97 (43.5) | 72 (43.6) | 25 (43.1) |  |
| Small vessel occlusion | 92 (41.3) | 69 (41.8) | 23 (39.7) |  |
| Cerebral small vessel disease |  |  |  |  |
| High-grade white matter hyperintensities | 56 (25.1) | 33 (20.0) | 23 (39.7) | 0.003 |
| Cerebral microbleeds | 45 (20.2) | 23 (13.9) | 22 (37.9) | <0.001 |
| High-grade perivascular spaces | 22 (9.9) | 7 (4.2) | 15 (25.9) | <0.001 |
| Asymptomatic lacunar infarctions | 48 (21.5) | 22 (13.3) | 26 (44.8) | <0.001 |
| Total small vessel disease score |  |  |  | <0.001 |
| 0 | 125 (56.1) | 104 (63.0) | 21 (36.2) |  |
| 1 | 56 (25.1) | 45 (27.3) | 11 (19.0) |  |
| 2 | 18 (8.1) | 8 (4.8) | 10 (17.2) |  |
| 3 | 17 (7.6) | 8 (4.8) | 9 (15.5) |  |
| 4 | 7 (3.1) | 0 (0.0) | 7 (12.1) |  |
| Blood laboratory findings |  |  |  |  |
| Plasma Klotho, pg/mL | 329.8 ± 194.1 | 354.3 ± 202.7 | 251.8 ± 146.3 | <0.001 |
| Fibroblast growth factor-23, pg/ml | 343.7 ± 546.2 | 263.4 ± 352.1 | 663.1 ± 930.1 | 0.002 |
| Vitamin D 25(OH)D, ng/mL | 20.1 ± 6.8 | 20.8 ± 7.2 | 19.2 ± 5.8 | 0.100 |
| Fasting glucose, mg/dL | 116.2 ± 42.3 | 115.1 ± 42.3 | 115.5 ± 36.0 | 0.957 |
| HbA1c, % | 6.5 ± 1.4 | 6.6 ± 1.4 | 6.3 ± 1.1 | 0.236 |
| Triglyceride, mg/dL | 129.0 ± 95.6 | 127.8 ± 92.1 | 137.1 ± 113.5 | 0.535 |
| Total cholesterol, mg/dL | 177.5 ± 38.9 | 181.1 ± 38.8 | 170.1 ± 39.2 | 0.067 |
| Low-density lipoprotein, mg/dL | 115.0 ± 36.9 | 117.6 ± 35.8 | 106.4 ± 40.2 | 0.047 |
| White blood cell count, ×10^3^ | 7.3 ± 2.4 | 7.2 ± 2.4 | 7.9 ± 2.7 | 0.089 |
| Haemoglobin, mg/dL | 13.5 ± 1.6 | 13.5 ± 1.6 | 13.6 ± 1.3 | 0.833 |
| Creatinine, mg/dL | 1.0 ± 0.8 | 0.9 ± 0.5 | 1.1 ± 0.9 | 0.140 |
| Total calcium, mg/dL | 8.2 ± 0.4 | 8.2 ± 0.4 | 8.3 ± 0.4 | 0.098 |
| Phosphate, mg/dL | 3.1 ± 0.6 | 3.1 ± 0.6 | 3.0 ± 0.7 | 0.153 |
| Albumin, mg/dL | 3.7 ± 0.3 | 3.7 ± 0.3 | 3.6 ±0.2 | 0.440 |
| Alkaline phosphatase, IU/L | 224.4 ± 72.2 | 221.5 ± 66.9 | 237.1 ± 83.1 | 0.154 |
| Uric acid, mg/dL | 4.8 ± 1.6 | 4.8 ± 1.5 | 4.7 ± 1.6 | 0.557 |
| C-reactive protein, mg/L | 0.9 ± 2.8 | 1.0 ± 3.2 | 1.0 ± 2.1 | 0.938 |
